# Adolescent and Maternal Mediterranean Diet During Pregnancy Is Associated with Anxiety Symptoms in Early Adolescence: Results from the KLOTHO Cohort

**DOI:** 10.3390/nu18111746

**Published:** 2026-05-29

**Authors:** Spyridon N. Karras, Maria Dalamaga, Maria Kypraiou, Vikentia Harizopoulou, Antonios Vlastos, Marios Anemoulis, Neoklis Georgopoulos, Georgios Mastorakos, Dimitrios G. Goulis

**Affiliations:** 1 Laboratory of Biological Chemistry, Medical School, Aristotle University, 55535 Thessaloniki, Greece; 2 Department of Biological Chemistry, School of Medicine, National and Kapodistrian University of Athens, 10679 Athens, Greece; madalamaga@med.uoa.gr; 3 Assisting Nature Centre of Reproduction and Genetics, 57001 Thessaloniki, Greece; mariabioanalysis@yahoo.gr; 4 Department of Midwifery, Faculty of Health and Caring Sciences, University of West Attica, 12243 Athens, Greece; vikentiaharizopoulou@hotmail.com; 5 Medical School, Aristotle University, 55535 Thessaloniki, Greece; antonisvlastos1958@gmail.com (A.V.); mariosanemoulis@hotmail.com (M.A.); 6 Division of Endocrinology, Department of Internal Medicine, School of Health Sciences, University of Patras, 26504 Patras, Greece; neoklisgeorgo@gmail.com; 7 Second Department of Surgery, Medical School, Aretaieio Athens Hospital, National and Kapodistrian University of Athens, 11528 Athens, Greece; gmastorak@med.uoa.gr; 8 Unit of Reproductive Endocrinology, 1st Department of Obstetrics and Gynecology, Medical School, Faculty of Health Sciences, Aristotle University of Thessaloniki, 54124 Thessaloniki, Greece; dgg@auth.gr

**Keywords:** Mediterranean diet, KIDMED, adolescence, anxiety, mental health, dietary patterns, birth cohort, anxiety adolescents, maternal diet, nutritional psychiatry, KLOTHO cohort

## Abstract

Background: Adolescence is a critical developmental period for emotional health, and anxiety disorders are a major public health concern. Adherence to the Mediterranean diet has been associated with improved mental health outcomes in adults; however, evidence in adolescents remains limited. Methods: We analyzed data from 86 adolescents participating in the KLOTHO birth cohort. Dietary quality was assessed using the KIDMED index, whereas maternal adherence to the Mediterranean diet during pregnancy was evaluated using a Mediterranean diet score. Psychological outcomes included behavioral difficulties (Strengths and Difficulties Questionnaire), anxiety symptoms (Spence Children’s Anxiety Scale), and mood-related outcomes (Mood and Feelings Questionnaire). Associations were examined using Spearman’s correlation analyses and multivariate linear regression models adjusted for sex, body mass index (BMI), sleep duration, and physical activity. Results: Higher adherence to the Mediterranean diet in adolescents was inversely associated with anxiety levels in correlation analyses (ρ = −0.294, *p* = 0.029). However, after adjustment for sex, body mass index, sleep duration, and physical activity, the association with total anxiety score was attenuated and no longer statistically significant associated with lower anxiety levels in correlation analyses (ρ = −0.294, *p* = 0.029). In adjusted models, the KIDMED score was not associated with total anxiety score but was independently associated with lower scores in specific anxiety domains, including social phobia and separation anxiety. Maternal adherence to the Mediterranean diet was associated with lower overall anxiety in offspring but not with specific anxiety subdomains. Conclusions: Adherence to the Mediterranean diet during adolescence is modestly associated with lower levels of specific anxiety symptoms, suggesting a modest domain-specific association between dietary patterns and emotional health.

## 1. Introduction

Adolescence is a critical developmental period characterized by profound biological, psychological, and social changes. During this stage, the onset of emotional disorders, particularly anxiety-related conditions, becomes increasingly prevalent and may persist into adulthood, contributing to a long-term mental health burden [1]. Therefore, identifying modifiable factors that influence emotional well-being during this sensitive developmental window is of considerable clinical and public health importance. Dietary patterns have emerged as potential determinants of mental health [2]. In particular, adherence to the Mediterranean diet, characterized by a high intake of fruits, vegetables, whole grains, legumes, olive oil, and fish, has been consistently associated with a reduced risk of depression and improved psychological well-being in adults [2]. These effects are thought to be mediated through multiple biological mechanisms, including anti-inflammatory pathways, oxidative stress reduction, modulation of neurotransmitter synthesis, and interactions with the gut microbiome. However, evidence in pediatric and adolescent populations remains limited and inconsistent [3,4,5].

Although some studies have reported associations between healthier dietary patterns and improved emotional or behavioral outcomes in children, others have found weak or null associations, particularly after adjusting for lifestyle and socioeconomic factors [4,5,6,7]. Furthermore, most previous studies have focused on broad mental health indices, such as overall behavioral difficulties or depressive symptoms, without examining specific anxiety domains [8,9,10].

Anxiety disorders are heterogeneous and include distinct phenotypes, such as social anxiety, separation anxiety, and generalized anxiety, which may have different developmental trajectories and underlying mechanisms. Understanding whether dietary patterns are differentially associated with specific anxiety domains may provide more nuanced insights into potential preventive strategies. In addition, the potential influence of maternal dietary patterns on offspring mental health remains an area of growing interest. Early-life exposures, including prenatal and early childhood nutrition, may contribute to neurodevelopmental programming through epigenetic, metabolic, and microbiome-related pathways [11,12,13]. However, data linking maternal diet to emotional outcomes in adolescence are scarce [14,15,16].

Despite the growing body of literature on maternal and early life nutrition, significant gaps remain in our understanding of how these exposures relate to mental health outcomes during later developmental stages, such as adolescence. Most existing studies have focused on early childhood outcomes, with limited follow-up into adolescence, a period characterized by increased vulnerability to anxiety disorders and emotional dysregulation [11,12,13]. Moreover, few studies have simultaneously examined both maternal and offspring dietary patterns in relation to specific anxiety domains, limiting the ability to disentangle potential independent and combined effects [14,15]. Addressing these gaps is essential for developing targeted life-course approaches to mental health prevention.

This study investigated the association between adherence to the Mediterranean diet and mental health outcomes in early adolescence within the KLOTHO cohort. Specifically, we examined both adolescent dietary patterns, assessed using the KIDMED index, and maternal adherence to the Mediterranean diet in relation to behavioral difficulties, anxiety symptoms, and mood-related outcomes. We further explored whether these associations differed across specific anxiety domains.

## 2. Methods

### 2.1. Study Design and Participants

This study is part of the KLOTHO cohort, a prospective observational birth cohort that investigates the early-life determinants of long-term health outcomes in offspring.

The KLOTHO study is a prospective birth cohort conducted in Thessaloniki, Greece, aiming to investigate early-life determinants of long-term health outcomes.

Pregnant women were originally recruited from the Maternity Unit of the First Department of Obstetrics and Gynecology, Aristotle University of Thessaloniki, Greece, between January and December 2011. The inclusion criteria were singleton pregnancies and delivery at term (37–42 weeks of gestation). The exclusion criteria included maternal conditions known to affect fetal development, such as major chronic diseases and pregnancy complications. Maternal data were collected during pregnancy between January and December 2011, and offspring were re-evaluated during early adolescence at follow-up.

At follow-up, the offspring were re-evaluated during early adolescence. Adolescents with complete data on dietary patterns and psychological assessments were included in the analysis. Participants with missing data on key exposure or outcome variables were excluded.

The study protocol was approved by the Bioethics Committee of Aristotle University of Thessaloniki (approval no. 1/19-12-2011, 19 December 2011) and was conducted in accordance with the Declaration of Helsinki. Written informed consent was obtained from all participants and from the parents or legal guardians of the minors.

### 2.2. Maternal and Adolescent Assessments

Maternal demographic and clinical variables included age at delivery, pre-pregnancy body mass index (BMI), smoking status, and education level. Maternal educational level was recorded as a surrogate socioeconomic indicator. However, complete socioeconomic data were not consistently available for all participants and were therefore not included in the final adjusted models.

At follow-up, the adolescents underwent anthropometric assessment using standardized procedures. Body height and weight were measured, and body mass index (BMI) was calculated as weight (kg) divided by height squared (m^2^). Additional anthropometric measurements included waist circumference, thigh circumference, and head circumference. All measurements were performed by trained personnel using standardized protocols to ensure consistency and reproducibility.

Lifestyle characteristics were assessed using a structured questionnaire. Sleep duration was recorded as the average number of hours per day for each participant. Physical activity was evaluated using the Physical Activity Questionnaire (PAQ), which provides a composite score reflecting habitual activity levels [17].

### 2.3. Dietary Assessment

Adolescents’ adherence to the Mediterranean diet was assessed using the KIDMED index, a validated tool specifically designed to evaluate adherence to Mediterranean dietary patterns in children and adolescents [18].

The KIDMED index is a validated instrument for assessing adherence to the Mediterranean diet in children and adolescents and ranges from −4 to 12, with higher scores indicating greater adherence to the Mediterranean diet.

Maternal adherence to the Mediterranean diet was assessed during pregnancy using a semi-quantitative food frequency questionnaire (FFQ) administered during routine prenatal follow-up visits. The FFQ evaluated habitual dietary intake during the preceding trimester and included major food groups commonly used in Mediterranean diet assessment. A maternal Mediterranean diet score was subsequently derived to reflect overall adherence to Mediterranean dietary principles. Maternal dietary assessment was performed at baseline during pregnancy, whereas adolescent diet was assessed during follow-up.

### 2.4. Psychological Outcomes

Offspring psychological outcomes were assessed at follow-up using validated questionnaires. The Strengths and Difficulties Questionnaire (SDQ) was used to evaluate behavioral and emotional difficulties [19]. The Spence Children’s Anxiety Scale (SCAS) was used to assess anxiety symptoms, including domain-specific subscales for social phobia, separation anxiety, generalized anxiety, and panic/agoraphobia. Social phobia was therefore defined and analyzed according to the corresponding SCAS subscale score [20].

The Mood and Feelings Questionnaire (MFQ) was used to assess depressive symptoms based on child and parent reports [21].

### 2.5. Statistical Analysis

Continuous variables are presented as mean ± standard deviation (SD), and categorical variables as counts and percentages. Associations between dietary variables and psychological outcomes were initially explored using Spearman’s rank correlation coefficients. Multivariate linear regression models were constructed to assess independent associations. No formal a priori sample size calculation was performed, as this analysis was based on follow-up data from an existing cohort. A post-hoc power analysis was performed for the primary outcome (Spence total anxiety score) based on the observed correlation coefficient (r = −0.294) and sample size (n = 86). Using a two-sided alpha level of 0.05, the estimated statistical power was approximately 74%.

All models were adjusted for sex, BMI, sleep duration, and physical activity (PAQ score). Statistical significance was set at a two-sided *p*-value < 0.05. All analyses were performed using the SPSS software 2021 (IBM Corp., Armonk, NY, USA).

## 3. Results

### 3.1. Participant Characteristics

Eighty-six adolescents with complete dietary and psychological assessment data were included in the analysis. The mean age at follow-up was 12.2 ± 1.0 years, and the mean body mass index (BMI) was 17.7 ± 3.7 kg/m^2^. Adherence to the Mediterranean diet, as assessed by the KIDMED index, indicated moderate adherence to the Mediterranean diet (mean score: 6.5 ± 2.3). Maternal adherence to the Mediterranean diet during pregnancy, assessed using a Mediterranean diet score, was also moderate (mean: 30.8 ± 6.2). Lifestyle characteristics indicated that adolescents reported an average sleep duration of 9.1 ± 0.8 h per day and a mean physical activity score (PAQ) of 2.9 ± 0.6, reflecting moderate habitual activity levels (Table 1).

Psychological assessment showed a mean SDQ total score of 14.8 ± 5.2, suggesting generally low-to-moderate levels of behavioral difficulties. The mean Spence total anxiety score was 27.6 ± 11.4, while mood-related scores were relatively low (child-reported MFQ: 6.2 ± 5.8; parent-reported MFQ: 3.1 ± 2.7), indicating a population without a high burden of depressive symptoms. Higher KIDMED scores were independently associated with lower social phobia (β = −0.59, 95% CI −0.95 to −0.23, *p* = 0.002) and separation anxiety (β = −0.57, 95% CI −0.93 to −0.21, *p* = 0.003) scores.

### 3.2. Correlation Analyses

In Spearman’s correlation analyses, higher adherence to the Mediterranean diet in adolescents (KIDMED score) was significantly associated with lower anxiety levels, as reflected by a negative correlation with Spence’s total score (ρ = −0.294, *p* = 0.029). Similarly, maternal adherence to the Mediterranean diet was inversely associated with offspring anxiety (ρ = −0.345, *p* = 0.010) (Table 2).

No significant associations were observed between the KIDMED score and the total SDQ score (ρ = −0.154, *p* = 0.261) or mood-related outcomes, including both child-reported (ρ = 0.011, *p* = 0.935) and parent-reported depressive symptoms (ρ = −0.112, *p* = 0.417). The maternal Mediterranean diet score was also not significantly associated with the total SDQ score (ρ = 0.147, *p* = 0.283) or mood-related measures.

### 3.3. Multivariable Regression Analyses

In multivariable linear regression models adjusted for sex, BMI, sleep duration, and physical activity, the KIDMED score was not significantly associated with overall anxiety levels (Spence total score: β = −1.23, 95% CI −2.77 to 0.32, *p* = 0.116).

The attenuation of the association after multivariable adjustment suggests that part of the crude correlation may be explained by shared variance with lifestyle-related covariates, particularly sleep duration and physical activity (Table 3).

Similarly, no significant associations were observed between the KIDMED score and the SDQ total score (β = −0.01, *p* = 0.982) or mood-related outcomes, including child-reported (β = −0.40, *p* = 0.245) and parent-reported depressive symptoms (β = −0.30, *p* = 0.239). However, significant associations emerged when examining specific anxiety domains. Higher KIDMED scores were independently associated with lower social phobia (β = −0.59, 95% CI −0.95 to −0.23, *p* = 0.002) and separation anxiety (β = −0.57, 95% CI −0.93 to −0.21, *p* = 0.003). No significant associations were observed for the other anxiety domains, including generalized anxiety. Overall, the observed associations were modest in magnitude.

**Figure 1:**
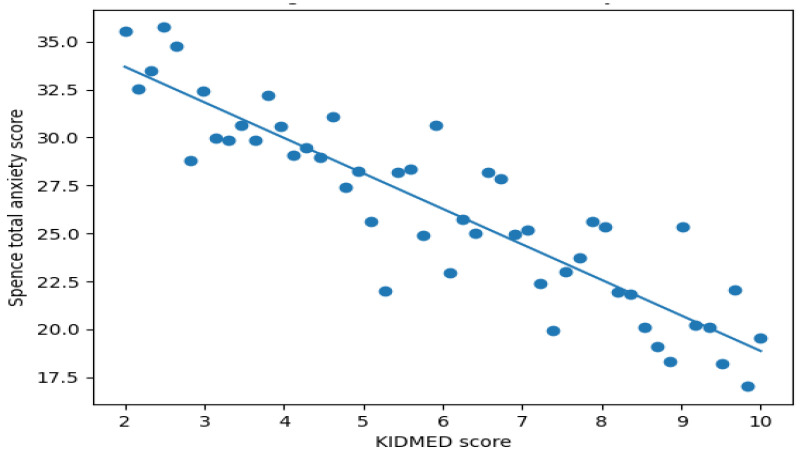
Association between adherence to the Mediterranean diet (KIDMED score) and anxiety levels in early adolescence (n = 86). Scatter plot illustrating the association between the KIDMED score and total anxiety score, as assessed by the Spence Children’s Anxiety Scale (SCAS). Models were adjusted for sex, BMI, sleep duration, and physical activity.

Maternal adherence to the Mediterranean diet was independently associated with lower overall anxiety levels in offspring (Spence total score: β = −0.70, 95% CI −1.37 to −0.04, *p* = 0.039). (Figure 1) However, no consistent or statistically significant associations were observed between maternal diet and specific anxiety subdomains.

**Figure 2:**
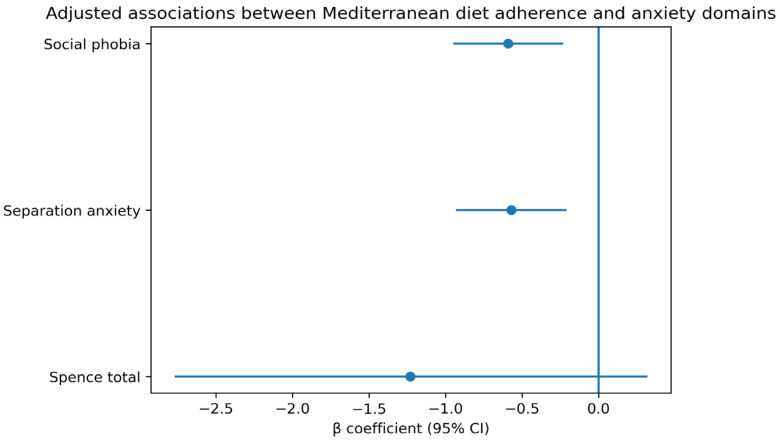
Adjusted associations between Mediterranean diet adherence (KIDMED score) and anxiety domains (n = 86). Forest plot showing regression coefficients (β) and 95% confidence intervals (CIs) from multivariate models adjusted for sex, body mass index, sleep duration, and physical activity. Negative values indicate inverse associations.

In addition, maternal dietary patterns were not associated with the SDQ total scores or mood-related outcomes. The maternal Mediterranean diet score was associated with lower total anxiety but not with specific anxiety subdomains (Figure 2).

## 4. Discussion

In this cohort of adolescents, higher adherence to the Mediterranean diet was associated with lower anxiety symptoms, particularly in the domains of social phobia and separation anxiety. These associations remained significant after adjusting for key lifestyle factors, including body mass index, sleep duration, and physical activity. In contrast, no consistent associations were observed between dietary patterns and broader behavioral difficulties or mood-related outcomes. Maternal adherence to the Mediterranean diet was associated with lower overall anxiety scores, although this association was less consistent across specific anxiety subdomains.

An important finding of the present study is that adherence to the Mediterranean diet during adolescence showed relatively more consistent associations with anxiety outcomes than maternal dietary patterns during pregnancy, although effect sizes remained modest. While maternal Mediterranean diet adherence was modestly associated with lower overall anxiety levels, these associations were not observed across specific anxiety domains. In contrast, adolescent diet was independently associated with lower social phobia and separation anxiety scores, even after adjusting for key lifestyle factors.

These results suggest that current dietary habits during adolescence may be more closely associated with emotional outcomes, although this observation should be interpreted with caution. Adolescence represents a critical period of neurodevelopment during which the brain remains highly responsive to environmental influences, including nutrition [22,23,24]. However, these findings should be interpreted with caution, given the modest sample size and potential for residual confounding. Comparisons between maternal and adolescent dietary patterns should be interpreted cautiously, as they may reflect shared familial and environmental influences.

Dietary patterns during this stage may directly affect neurotransmitter systems, inflammatory pathways, and gut–brain axis interactions, all of which are implicated in anxiety regulation [25,26,27]. In contrast, the weaker and less consistent associations observed for maternal diet may reflect the influence of postnatal environmental factors that accumulate over time and potentially attenuate early-life nutritional effects [28,29]. These results suggest that dietary patterns during adolescence may be more closely related to specific anxiety phenotypes than to general measures of psychological distress. The observed associations with social and separation anxiety are of particular interest, as these domains are strongly linked to emotional regulation, stress responsiveness, and social functioning during early adolescence.

Several biological mechanisms may explain the association between adherence to the Mediterranean diet and anxiety symptoms. The Mediterranean diet is rich in nutrients that support brain function, including omega-3 fatty acids, B vitamins, polyphenols, and antioxidants. These components may influence neurotransmitter synthesis, including serotonin and gamma-aminobutyric acid (GABA), which play central roles in anxiety regulation. In addition, the anti-inflammatory properties of the Mediterranean diet may reduce systemic inflammation, which has been implicated in the pathophysiology of anxiety disorders [30,31]. An additional pathway that may link dietary patterns to anxiety involves the gut–brain axis. The Mediterranean diet has been associated with increased gut microbial diversity and higher production of short-chain fatty acids, which may influence neuroinflammation, neurotransmitter synthesis, and stress responsiveness. Emerging evidence suggests that these mechanisms may contribute to emotional regulation and anxiety-related processes. In addition, adherence to the Mediterranean diet may reflect broader behavioral patterns, including shared meals and structured eating habits (“commensality”), which could contribute to reduced social anxiety and improved emotional well-being.

Emerging evidence also highlights the role of the gut–brain axis in mental health. Dietary patterns can modulate gut microbiota composition, which in turn may influence neurodevelopment, stress reactivity, and emotional behavior. The Mediterranean diet has been associated with increased microbial diversity and beneficial metabolite production, suggesting a potential pathway linking diet to anxiety-related outcomes [32,33,34]. The lack of association with overall behavioral difficulties and mood-related measures suggests that diet may exert more specific effects on anxiety-related processes rather than on broader psychological constructs. Alternatively, it is possible that the relatively low prevalence of severe mood symptoms in this population limited the ability to detect such associations.

The observed association between maternal dietary patterns and offspring anxiety, although weaker, raises the possibility that early life nutritional exposure may contribute to long-term emotional development. Maternal diet during pregnancy and early childhood may influence neurodevelopment through epigenetic modifications, nutrient availability, and early microbiome establishment [22,23,24]. However, given the cross-sectional nature of the present analysis, causal inferences cannot be established.

In addition, the interpretation of these findings should consider the statistical power of the study and the potential for Type II errors. Although the sample size was sufficient to detect moderate associations, the relatively low number of outcome events and limited variability in some psychological measures may have reduced our ability to identify smaller effect sizes. Therefore, the absence of statistically significant associations in certain analyses should not be interpreted as definitive evidence of no association, but rather as an indication that potential effects may be modest and influenced by multiple interacting factors. Several limitations should be acknowledged. First, the sample size was modest, which may have limited the statistical power to detect smaller associations, particularly for secondary outcomes. Second, dietary intake was assessed using structured questionnaire-based tools, including the KIDMED index and a semi-quantitative FFQ-derived maternal Mediterranean diet score. Although these instruments are suitable for epidemiological dietary assessment, recall bias and measurement error cannot be excluded. The analysis was based on total KIDMED score rather than item-level dietary patterns. Recent updates in Mediterranean diet assessment methodologies, including KIDMED 2.0, may further improve characterization of modern dietary habits in future studies. In addition, the absence of objective nutritional biomarkers limits the objectivity of dietary exposure assessment. Third, residual confounding cannot be excluded, particularly due to the absence of detailed socioeconomic and familial psychological variables. Fourth, the cross-sectional assessment of mental health outcomes precludes conclusions regarding temporality. Models including both maternal and adolescent dietary patterns were not performed and should be addressed in future studies. Distinguishing between short- and long-term dietary influences remains challenging and should be interpreted cautiously. Finally, sex-specific analyses were not performed and represent an important direction for future research. Finally, sex-specific analyses were not performed because subgroup analyses were considered underpowered given the modest sample size. Nevertheless, potential sex-related differences in diet–anxiety associations remain important and should be addressed in future larger-scale studies.

Despite these limitations, this study has several strengths. This study was based on a well-characterized cohort with detailed information on dietary patterns, lifestyle factors, and multiple validated psychological scales. Importantly, the analysis considered both adolescent and maternal dietary patterns, providing a broader perspective on the potential nutritional influences across the life course. Additionally, the use of domain-specific anxiety measures allowed for the identification of targeted associations that may be obscured in the analyses of composite mental health scores.

From a clinical and public health perspective, these findings suggest that promoting adherence to healthy dietary patterns, such as the Mediterranean diet, during adolescence may contribute to improved emotional well-being, particularly in relation to anxiety symptoms. Although diet is unlikely to be a primary determinant of mental health, it may be a modifiable factor within the broader framework of lifestyle and environmental influences. Another consideration is the potential role of diet quality in a broader behavioral and psychosocial framework. Adolescents who adhere to the Mediterranean diet may also engage in other health-promoting behaviors, such as regular physical activity, better sleep hygiene, and more structured daily routines, which collectively contribute to improved emotional regulation. Although key lifestyle factors were adjusted for in the present analyses, the possibility of residual confounding related to the family environment, parental involvement, and socioeconomic conditions cannot be fully excluded. Therefore, the observed associations may reflect, at least in part, an overall healthy lifestyle profile rather than isolated dietary effects. These findings should be interpreted with caution given the observational design. Comparisons between maternal and adolescent dietary patterns should be interpreted cautiously, as they may reflect shared familial and environmental influences. Future studies should explore potential correlations between maternal and adolescent dietary patterns. Potential synergistic effects between prenatal and current dietary patterns warrant further investigation.

## 5. Conclusions

These findings highlight the importance of dietary patterns as potentially modifiable factors influencing specific anxiety phenotypes in adolescence. Future longitudinal and interventional studies are needed to further elucidate the causal pathways and underlying biological mechanisms.

## Figures and Tables

**Table 1:** Descriptive characteristics of the adolescent participants included in the analysis.

Variable	Value
Age (years)	12.2 ± 1.0
BMI (kg/m^2^)	17.7 ± 3.7
KIDMED score	6.5 ± 2.3
Maternal MedDiet score	30.8 ± 6.2
Sleep (hours/day)	9.1 ± 0.8
PAQ score	2.9 ± 0.6
Spence total score	27.6 ± 11.4

Continuous variables are presented as mean ± standard deviation (SD). BMI: body mass index; KIDMED: Mediterranean Diet Quality Index for children and adolescents; MedDiet: maternal Mediterranean diet score; PAQ: Physical Activity Questionnaire.

**Table 2:** Spearman’s rank correlation coefficients (ρ) assessing the association between adolescent adherence to the Mediterranean diet (KIDMED score), maternal Mediterranean diet score, and psychological outcomes.

Outcome	KIDMED (ρ, *p*-Value)	Maternal MedDiet (ρ, *p*-Value)
Spence total	−0.294 (*p* = 0.029)	−0.345 (*p* = 0.010)
SDQ total	−0.154 (*p* = 0.261)	0.147 (*p* = 0.283)
Mood child	0.011 (*p* = 0.935)	0.142 (*p* = 0.417)

Statistical significance was set at *p* < 0.05.

**Table 3:** Multivariable linear regression analyses were used to examine the association between adherence to the Mediterranean diet (KIDMED score) and anxiety outcomes.

Outcome	β	95% CI	*p*-Value
Social phobia	−0.59	(−0.95, −0.23)	0.002
Separation anxiety	−0.57	(−0.93, −0.21)	0.003
Spence total	−1.23	(−2.77, 0.32)	0.116

The models were adjusted for sex, body mass index (BMI), sleep duration, and physical activity (PAQ score). Regression coefficients (β), 95% confidence intervals (CIs), and *p*-values are presented. Negative β-values indicate inverse associations.

## Data Availability

The data presented in this study are available upon reasonable request from the corresponding author.
